# Paeoniflorin ameliorates interferon-alpha-induced neuroinflammation and depressive-like behaviors in mice

**DOI:** 10.18632/oncotarget.14160

**Published:** 2016-12-24

**Authors:** Jianwei Li, Shaohui Huang, Weiliang Huang, Wanshan Wang, Ge Wen, Lei Gao, Xiuqiong Fu, Mengmeng Wang, Weihai Liang, Hiu Yee Kwan, Xiaoshan Zhao, Zhiping Lv

**Affiliations:** ^1^ School of Traditional Chinese Medicine, Southern Medical University, Guangzhou, Guangdong, 510515, China; ^2^ Experimental Animal Center, Southern Medical University, Guangzhou, Guangdong, 510515, China; ^3^ Medical Imaging Department, Nanfang Hospital, Southern Medical University, Guangzhou, Guangdong, 510515, China; ^4^ School of Chinese Medicine, Hong Kong Baptist University, Hong Kong, 999077, China

**Keywords:** depression, interferon, amygdala, neuroinflammation, paeoniflorin

## Abstract

Long-term treatment with high-dose Interferon-alpha (IFN-α) has resulted in depression in 30–50% of the patients. Paeoniflorin may ameliorate the IFN-α-induced depression; however, the underlying mechanism is less studied. Here, we investigated the prophylactic antidepressant and anti-neuroinflammatory effects of paeoniflorin on the behaviors and specific emotion-related regions of the brain in mice with IFN-α-induced depression. A series of behavior assessments were conducted to identify the depressive state after subcutaneously IFN-α injections and with or without intragastrically paeoniflorin administration in C57BL/6J mice. Levels of many inflammatory-related cytokines in serum, mPFC, vHi and amygdala were determined by cytokine array analysis. Furthermore, microglia and astrocyte activation in these three regions were evaluated by immunohistochemistry. We found that the mice which were subcutaneously injected IFN-α 15×10^6^ IU/kg for 4 successive weeks to mimic an IFN-α-induced depression model had distinct inflammatory changes in the amygdala. Interestingly, 4-week 20 mg/kg or 40 mg/kg paeoniflorin pretreatments reversed the depressive-like behaviors and the abnormal inflammatory cytokine levels in the serum, mPFC, vHi and amygdala. These cytokines were not limited to the commonly reported IL-6, IL-1β and TNF-α, but also IL-9, IL-10, IL-12, and MCP-1. Besides, the increased density of microglia in IFN-α-treated mice was reversed by paeoniflorin in these three brain areas. Taken together, our data suggest that paeoniflorin can reverse the long-term, high-dose IFN-α-induced depressive-like behaviors that were associated with local distinct neuroinflammation in the mPFC, vHi and particularly the amygdala. Paeoniflorin might have a preventive therapeutic potential in IFN-α-induced depression.

## INTRODUCTION

Interferon-alpha (IFN-α) is a pleiotropic cytokine with antiviral and antiproliferative effects and is widely used in the treatment of cancers and chronic viral hepatitis, including malignant melanoma and hepatitis C [1]. However, approximately 30–50% of patients (depending on dose) after receiving IFN-α therapy develop IFN-α-induced depression, with symptoms consistent with the *Diagnostic and Statistical Manual of Mental Disorders, Fourth Edition* criteria for major depression [2–4]. This can result in early discontinuation of the IFN-α treatment and therefore hinders its clinical application. Thus, a prophylactic antidepressant is necessary [5, 6].

The use of prophylactic antidepressants has been supported by a recent systematic review and meta-analysis of chronic hepatitis C patients with IFN-α-induced depression, which demonstrated a significant preventive effect of selective serotonin reuptake inhibitors (SSRIs), especially escitalopram [7]. However, not all SSRIs have significant preventive effect in patients with hepatitis C virus infection who need to receive IFN-α treatment [8–10]. Moreover, the potential for SSRIs to induce dizziness and gastrointestinal bleeding is of particular concern for patients [7, 11]. Furthermore, some rare but severe side effects, such as renal injury, cotton-wool spots, and manic episodes, have been observed in patients who have undergone SSRIs administration [12, 13]. Therefore, it is important to find alternative strategies to ameliorate IFN-α-induced depression.

A suggested mechanism underlying the pathogenesis of IFN-α-induced depression is mediated by inflammatory cytokines in the brain, especially in the emotion-related regions such as the prefrontal cortex and hippocampus, which result in depressive-like behaviors [14]. It is well-known that the medial prefrontal cortex (mPFC) performs a key function in processing convergent cognitive and emotionally relevant information, and this has been reported to be correlated with IFN-α-induced depression [15–17]. In addition, the ventral hippocampus (vHi) is preferentially implicated in emotion, stress and anxiety, which plays an important role in depressive disorders [18, 15]. Moreover, IFN-α is a small polypeptide that is able to access the brain parenchyma when systemically administered and induce the activation of a broad set of cytokines and chemokines in the brain, including interleukin (IL)-1β, IL-6, and tumor necrosis factor (TNF)-α [19, 20, 17]. It has recently been reported that cytokines and active microglia in the hippocampus might be associated with depressive-like behaviors in IFN-α-treated mice [21]. Furthermore, cytokines in the brain are produced not only by microglia but also by astrocytes, which suggests that microglia or astrocytes in some brain regions play a role in this subset of depression [22–24]. Furthermore, the amygdala seems to be another key player in fear learning, emotion, stress, and anxiety, since genes expressed in the vHi correlate with amygdala [18, 15]. However, little attention has been focused on the important region of the amygdala in terms of evaluating inflammatory-associated changes with respect to cytokines and chemokines, and its related microglia and astrocytes, in IFN-α-induced depression.

To address the above mentioned potential neuroinflammatory-associated mechanism, it seems that an alternative strategy for preventing IFN-α-induced depression should involve a prophylactic antidepressant with anti-neuroinflammatory effect. Paeoniflorin, an amorphous glucoside, is the main active component of total glycosides found in the root of the peony (*Paeonialactiflora* Pall) it exerts potential preventive and therapeutic effects against IFN-α-induced depression. The peony is one of the most commonly used drugs in Chinese herbal formulae for the treatment of depressive-like behaviors [26–28, 25]. As an important component of the peony, paeoniflorin significantly increases sucrose consumption and reverses the reductions of serotonin and its metabolite 5-hydroxyindoleacetic acid in a rat model of chronic unpredictable stress [29]. Moreover, paeoniflorin markedly reduces the immobility time in forced swimming tests (FSTs) and tail suspension tests (TSTs) when intraperitoneally injected into mouse models [30]. In addition, paeoniflorin significantly blocks the lipopolysaccharide-induced hippocampal cell death and the production of nitric oxide and IL-1β in hippocampal slice cultures, as well as in primary microglia cells [31]. Indeed, many reports suggest that paeoniflorin exhibits potential neuroprotective, anti-ischemic, antioxidative and anti-inflammatory effects [32–37]. However, little is known about paeoniflorin's antidepressant effect and its anti-neuroinflammatory effect on IFN-α-induced depression in animal model. Here, we suggest that paeoniflorin might be an effective prophylactic strategy in IFN-α-induced depression, which is considered to be a cytokine-induced subset of depression. Based on its reported anti-inflammatory ability, it is possible that paeoniflorin reduces the systemic IFN-α-increased proinflammatory cytokines and inhibits the activation of immunocytes in the brain.

In light of the above, we hypothesized that paeoniflorin might ameliorate IFN-α-induced depressive-like behaviors in mice by influencing cytokine release and activation of microglia or astrocytes to attenuate neuroinflammation in critical emotion-related regions, such as the mPFC, vHi, and particularly the amygdala. The prophylactic antidepressant-like and anti-inflammatory effects of paeoniflorin and their effects on microglia and astrocytes in the mPFC, vHi, and amygdala were assessed using behavioral tests, cytokine array analysis and immunofluorescence.

## RESULTS

### Identification of a suitable IFN-α dosage

**Figure 1:**
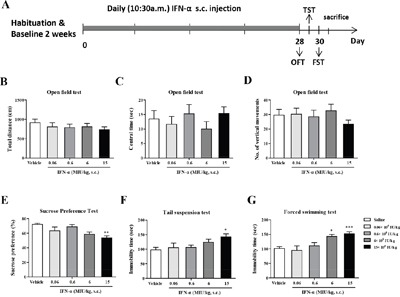
Identification of the interferon (IFN)-α dosage required to establish a model of IFN-α-induced depression in mice Experimental design. **A**. The effects of four different doses of IFN-α on depressive-like behaviors in mice. **B**–**G**. After the 28day injections of IFN-α, mice were subjected to the open field test (B–D), the sucrose preference test (E), the tail suspension test (F), and the forced swimming test (G). The total distance covered, central time, and numbers of vertical movements were measured in the open field test to identify the locomotive activities of IFN-α-treated mice. The sucrose preference (%) was detected to reflect anhedonia, the core symptom in depression. The immobility times in the tail suspension test and forced swimming test were examined to evaluate despair behavior. The results of these tests were quantified and compared among the groups. n=10 mice per group. *p< 0.05, **p< 0.01, ***p< 0.001. Error bars: means ± SEM.

To determine a suitable dosage of IFN-α to mimic an IFN-α-induced depression mouse model, we performed a series of behavioral tests on the first set of mice after giving mice four different dosages of IFN-α (0.06–15×10^6^ IU/kg, s.c.) for 4 successive weeks. Significant decrease can be seen in sucrose preference test (one-way ANOVA, *F*_4,41_=5.48, *P*<0.01, followed by an post-hoc test, Tukey's tests, *P*<0.01, compared with vehicle group) and elevations in immobility time in both the FST (one-way ANOVA, *F*_4,30_=8.27, *P*<0.001, followed by an post-hoc test, Tukey's tests, *P*<0.001, compared with vehicle group) and TST (one-way ANOVA, *F*_4,28_=3.09, *P*<0.05, followed by an post-hoc test, Tukey's tests, *P*<0.05, compared with vehicle group) (typical depressive-like behavioral phenotypes in rodents) were noted following 4 weeks of 15×10^6^ IU/kg IFN-α treatments. In addition, a significantly increased immobility time in the FST (ANOVA followed by an post-hoc test, Tukey's tests, *P*<0.05) but not in the TST (ANOVA followed by an post-hoc test, Tukey's tests, *P*>0.05) was observed in the 6×10^6^ IU/kg IFN-α group. No significant change in locomotion activity in the open-field test (include Total distance, one-way ANOVA, *F*_4,40_=0.55, *P*>0.05; numbers of vertical movements, one-way ANOVA, *F*_4,43_=0.72, *P*>0.05; the central time, one-way ANOVA, *F*_4,43_=0.72, *P*>0.05) was observed with the other IFN-α dosages. Results of these three behavior assessments indicated that a dose of 15×10^6^ IU/kg IFN-α s.c. injection for 4 weeks was suitable to elicit depressive-like behaviors in mouse model (Figure 1).

### Paeoniflorin ameliorates IFN-α-induced depressive-like behaviors

**Figure 2:**
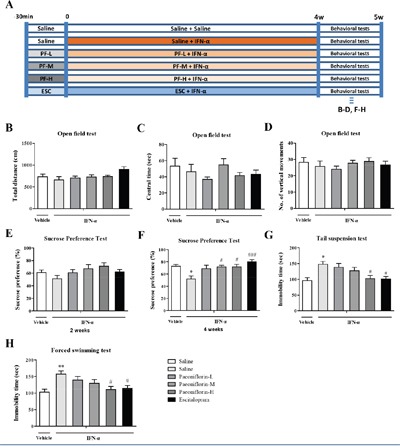
Paeoniflorin reversed interferon (IFN)-α-induced depressive-like behaviors in vivo Experimental design. **A**. The antidepressant effects of dose-dependent paeoniflorin on IFN-α treated mice for 4weeks. **B**–**H**. Mice were pretreated with paeoniflorin 30 min before IFN-α injection for 4weeks. After the 28day IFN-α injection, the mice were subjected to the open field test to evaluate locomotive activity by total distance covered (B), central time (C), and number of vertical movements (D). After 2 weeks of IFN-α administration in the middle period of study, the sucrose preference test (E) was performed to identify whether paeoniflorin or escitalopram had already had an effect; neither was found to have a significant antidepressant effect at this time. After 4 weeks, the sucrose preference test (F), the tail suspension test (G), and the forced-swimming test (H) produced significantly different results in paeoniflorin-treated mice, particularly in the high-dose group, as well as with escitalopram, a commonly used agent for treating depression. n=10 mice per group. *p< 0.05,**p< 0.01,***p< 0.001, compared with the saline-treated group; #p< 0.05, ##p< 0.01, ###p< 0.001 compared with the IFN-α-treated group. Error bars: means±SEM.

A low (10 mg/kg), medium (20 mg/kg), or high (40 mg/kg) dose of paeoniflorin or 10 mg/kg escitalopram was administered daily 30 min before IFN-α treatments for 4 weeks in order to examine the prophylactic antidepressant effects of the long-term high-dose IFN-α treatment in mice. Pretreatment of mice with high-dose paeoniflorin significantly reversed the increased immobility time elicited by IFN-α in both the FST (one-way ANOVA, *F*_5,44_=4.17, *P*<0.01, followed by an post-hoc test, Tukey's tests, *P*<0.05, compared with the model group. Figure 2H) and TST (one-way ANOVA, *F*_5,44_=4.53, *P*<0.01, followed by an post-hoc test, Tukey's tests, *P*<0.05, compared with the model group. Figure 2G). The SPT reflects anhedonia-like behavior, the core symptom of depression, and was conducted in the middle and at the end of the 4-week administration period to evaluate the effect of paeoniflorin within the treatment period. Furthermore, mice after receiving 2 weeks of pretreatment with high-dose paeoniflorin, the percentage of sucrose consumption was increased compared with the saline-treated group and the escitalopram group, although this failed to reach statistical significance (one-way ANOVA, *F*_5,43_=1.69, *P*>0.05. Figure 2E). At the end of the 4-week treatment period, medium- and high-dose paeoniflorin reversed the anhedonic effect caused by IFN-α in the SPT (one-way ANOVA, *F*_5,44_=4.65, *P*<0.01, followed by an post-hoc test, Tukey's tests, *P*<0.05, both the model group vs. medium dose paeoniflorin group and the model group vs. high dose group). The sucrose consumption rate was increased and reached the same level as seen with escitalopram, a commonly used antidepressant (Figure 2F). However, paeoniflorin treatment did not change the locomotive activity of the mice in the open field test (include Total distance, one-way ANOVA, *F*_5,44_=2.27, *P*>0.05; numbers of vertical movements, one-way ANOVA, *F*_5,44_=0.57, *P*>0.05; the central time, one-way ANOVA, *F*_5,44_=1.16, *P*>0.05. Figure 2B–2D). Overall, these behavioral changes indicated that paeoniflorin, especially at a high dose, exerted a prophylactic antidepressant effect in mice after receiving long-term high-dose IFN-α treatment.

### Paeoniflorin attenuates IFN-α-induced production of inflammation-associated cytokines in the serum

**Figure 3:**
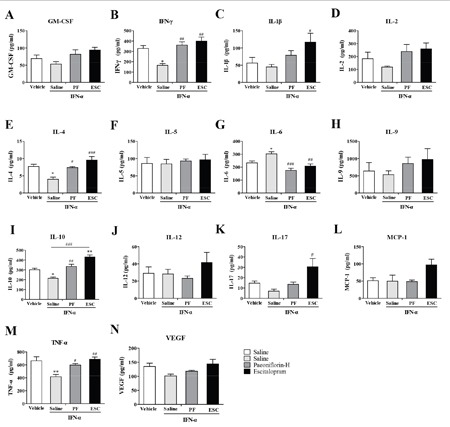
Anti-neuroinflammatory effects of paeoniflorin in serum The mice were sacrificed after 4weeks of treatment with paeoniflorin and behavioral tests. The serum was quickly removed and the levels of a series of inflammation-related cytokines and chemokines were analyzed using cytokine array analysis. The most effective dose of paeoniflorin in reversing depressive-like behaviors in interferon-α-treated mice, 40 mg/kg, showed the changed from IFN-α reversed in the serum. n=4 mice per group. *p<0.05, **p<0.01, ***p<0.001 compared with the saline-treated group; #p<0.05, ##p < 0.01, ###p<0.001 compared with the interferon-α-treated group. Error bars: means ± SEM.

Systemically administrated IFN-α can access the brain region from the periphery. To explore the antidepressant and anti-inflammatory mechanism of high-dose paeoniflorin, levels of the IFN-α-induced inflammatory related cytokines were detected in the serum. The levels of IFN-α (one-way ANOVA, *F*_3,11_=9.00, *P*<0.01, followed by an post-hoc test, Tukey's tests, *P*<0.05, compared with the vehicle group), IL-4(one-way ANOVA, *F*_3,11_=10.37, *P*<0.01, followed by an post-hoc test, Tukey's tests, *P*<0.05, compared with the vehicle group), IL-10 (one-way ANOVA, *F*_3,12_=24.38, *P*<0.001, followed by an post-hoc test, Tukey's tests, *P*<0.05, compared with the vehicle group) and TNF-α (one-way ANOVA, *F*_3,12_=8.77, *P*<0.01, followed by an post-hoc test, Tukey's tests, *P*<0.01, compared with the vehicle group) were decreased but IL-6 (one-way ANOVA, *F*_3,12_=11.40, *P*<0.001, followed by an post-hoc test, Tukey's tests, *P*<0.05, compared with the vehicle group) level was increased in the serum of mice after high dose IFN-α treatment. Paeoniflorin and escitalopram reversed these changes (Tukey's tests, *P*<0.05, compared with the model group. Figure 3).

### Paeoniflorin attenuates IFN-α-induced production of inflammation-associated cytokines in the medial prefrontal cortex

**Figure 4:**
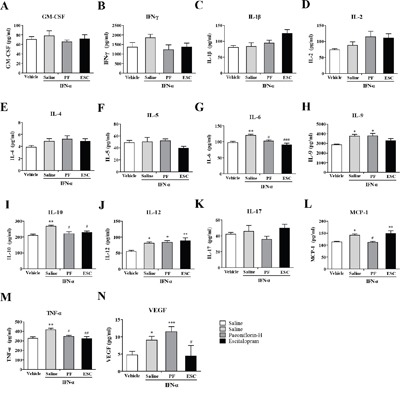
Anti-neuroinflammatory effects of paeoniflorin in the medial prefrontal cortex (mPFC) The mice were sacrificed after 4weeks of treatment with paeoniflorin and behavioral tests. The medial prefrontal cortex was quickly removed and the levels of a series of inflammation-related cytokines and chemokines were analyzed using cytokine array analysis. The most effective dose of paeoniflorin in reversing depressive-like behaviors in interferon-α-treated mice, 40 mg/kg, showed anti-neuroinflammatory effects in the emotion-related mPFC that were, in some instances, similar to those seen with escitalopram. n=4 mice per group. *p< 0.05, **p< 0.01, ***p< 0.001 compared with the saline-treated group; #p< 0.05, ##p < 0.01, ###p< 0.001 compared with the interferon-α-treated group. Error bars: means±SEM.

To further investigate the antidepressant mechanism of high-dose paeoniflorin, levels of IFN-α-induced inflammation-related cytokines were assessed in the mPFC, an emotion-associated brain region. The levels of IL-6 (one-way ANOVA, *F*_3,12_=9.99, *P*<0.01, followed by an post-hoc test, Tukey's tests, *P*<0.01, compared with the vehicle group), IL-9 (one-way ANOVA, *F*_3,12_=5.07, *P*<0.05, followed by an post-hoc test, Tukey's tests, *P*<0.05, compared with the vehicle group), IL-10 (one-way ANOVA, *F*_3,12_=7.53, *P*<0.01, followed by an post-hoc test, Tukey's tests, *P*<0.01, compared with the vehicle group), IL-12 p70 (one-way ANOVA, *F*_3,12_=6.15, *P*<0.01, followed by an post-hoc test, Tukey's tests, *P*<0.05, compared with the vehicle group), MCP-1 (one-way ANOVA, *F*_3,12_=8.83, *P*<0.01, followed by an post-hoc test, Tukey's tests, *P*<0.05, compared with the vehicle group), TNF-α (one-way ANOVA, *F*_3,12_=7.34, *P*<0.01, followed by an post-hoc test, Tukey's tests, *P*<0.01, compared with the vehicle group), and vascular endothelial growth factor (VEGF) (one-way ANOVA, *F*_3,12_=13.95, *P*<0.001, followed by an post-hoc test, Tukey's tests, *P*<0.05, compared with the vehicle group) were increased in mPFC when mice were treated with IFN-α. Paeoniflorin and escitalopram decreased most significantly, including IL-6 (Tukey's tests, *P*<0.05, paeoniflorin vs. model group; Tukey's tests, *P*<0.01, escitalopram vs. model group), IL-10 (Tukey's tests, *P*<0.05, both paeoniflorin vs. model group and escitalopram vs. model group), and TNF-α (Tukey's tests, *P*<0.05, paeoniflorin vs. model group; Tukey's tests, *P*<0.01, escitalopram vs. model group). However, there was no significant change in the increased IL-9 (*P*>0.05) level with either paeoniflorin or escitalopram treatment. In addition, mice treated with paeoniflorin had higher levels of IL-12 p70 and VEGF (*P*>0.05) than the vehicle control group. A similar phenomenon was observed with the IL-12 p70 level in the escitalopram group. Interestingly, the level of VEGF was significantly decreased with escitalopram (Tukey's tests, *P*<0.05). Furthermore, the MCP-1 level was higher than the vehicle group after escitalopram treatment (Tukey's tests, *P*<0.05) but paeoniflorin showed lower than that in the saline-treated group (Tukey's tests, *P*<0.05). Finally, escitalopram, but not paeoniflorin, significantly increased the IL-1β level compared with vehicle control group (Tukey's tests, *P*<0.05. Figure 4). These results suggest that paeoniflorin and escitalopram do have anti-inflammatory effects by regulation of cytokine production in the mPFC.

### Paeoniflorin attenuates IFN-α-induced production of inflammation-associated cytokines in the ventral hippocampus

**Figure 5:**
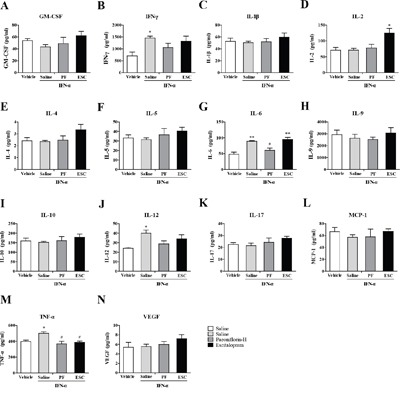
Anti-neuroinflammatory effects of paeoniflorin in the ventral hippocampus (vHi) The mice were sacrificed after 4 weeks of treatment with paeoniflorin and behavioral tests. The ventral hippocampus was quickly removed and the levels of a series of inflammation-related cytokines and chemokines were analyzed using cytokine array analysis. The most effective dose of paeoniflorin in reversing depressive-like behaviors in interferon-α-treated mice, 40 mg/kg, showed some anti-neuroinflammatory effects in the emotion-associated vHi, in part similar to those seen with escitalopram. n=4 mice per group. *p< 0.05, **p< 0.01, ***p< 0.001 compared with the saline-treated group; #p < 0.05, ##p < 0.01, ###p<0.001 compared with the interferon-α-treated group. Error bars: means±SEM.

Another major emotion-related region in the brain is the vHi. We used the same methods as described above to detect IFN-α-induced inflammation-related cytokines in vHi. The levels of IL-6 (one-way ANOVA, *F*_3,8_=15.47, *P*<0.01, followed by an post-hoc test, Tukey's tests, *P*<0.01, compared with the vehicle group), IL-12 p70(one-way ANOVA, *F*_3,12_=4.63, *P*<0.05, followed by an post-hoc test, Tukey's tests, *P*<0.05, compared with the vehicle group), TNF-α (one-way ANOVA, *F*_3,8_=7.13, *P*<0.05, followed by an post-hoc test, Tukey's tests, *P*<0.05, compared with the vehicle group), and IFN-γ (one-way ANOVA, *F*_3,12_=3.84, *P*<0.05, followed by an post-hoc test, Tukey's tests, *P*<0.05, compared with the vehicle group) were increased in the vHi after IFN-α treatment. Paeoniflorin decreased the reported increased levels of IL-6 (Tukey's tests, *P*<0.05) and TNF-α (Tukey's tests, *P*<0.05), but had no effect on the elevated levels of IL-12 p70 (Tukey's tests, *P*>0.05) and IFN-γ (Tukey's tests, *P*>0.05). Like paeoniflorin, escitalopram reversed the elevated TNF-α (Tukey's tests, *P*<0.05) level but had no effect on the elevated IL-6 (Tukey's tests, *P*>0.05) level caused by IFN-α which was even higher than in the vehicle group. In addition, the IL-2 level (Tukey's tests, *P*<0.05) was higher in mice pretreated with escitalopram than in that in the vehicle group (Figure 5).

### Paeoniflorin attenuates IFN-α-induced production of inflammation-associated cytokines in the amygdale

**Figure 6:**
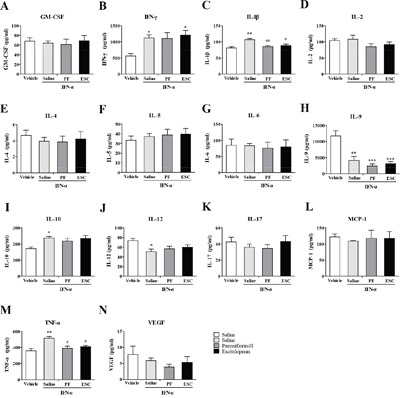
Anti-neuroinflammatory effects of paeoniflorin in the amygdala The mice were sacrificed after 4 weeks of treatment with paeoniflorin and behavioral tests. The amygdala was quickly removed and the levels of a series of inflammation-related cytokines and chemokines were analyzed using cytokine array analysis. The most effective dose of paeoniflorin in reversing depressive-like behaviors in interferon-α-treated mice, 40 mg/kg, showed some anti-neuroinflammatory effects in the emotion-associated amygdala, in part similar to those seen with escitalopram. n=4 mice per group. *p< 0.05, **p< 0.01, ***p< 0.001 compared with saline-treated group; #p< 0.05, ##p< 0.01, ###p< 0.001 compared with the interferon-α-treated group. Error bars: means ± SEM.

In addition to the mPFC and vHi, the amygdala also plays an important role in emotion. In order to further investigate the antidepressant mechanism of high-dose paeoniflorin, levels of the same cytokines were analyzed in the amygdala. The levels of IL-1β (one-way ANOVA, *F*_3,12_=8.30, *P*<0.01, followed by an post-hoc test, Tukey's tests, *P*<0.01, compared with the vehicle group), IL-10 (one-way ANOVA, *F*_3,12_=4.00, *P*<0.05, followed by an post-hoc test, Tukey's tests, *P*<0.05, compared with the vehicle group), TNF-α (one-way ANOVA, *F*_3,12_=8.42, *P*<0.01, followed by an post-hoc test, Tukey's tests, *P*<0.01, compared with the vehicle group), and IFN-γ (one-way ANOVA, *F*_3,12_=5.14, *P*<0.05, followed by an post-hoc test, Tukey's tests, *P*<0.05, compared with the vehicle group) in the amygdala were increased with IFN-α treatment, while the levels of IL-9 (one-way ANOVA, *F*_3,12_=15.61, *P*<0.001, followed by an post-hoc test, Tukey's tests, *P*<0.01, compared with the vehicle group) and IL-12p 70 (one-way ANOVA, *F*_3,12_=3.73, *P*<0.05, followed by an post-hoc test, Tukey's tests, *P*<0.05, compared with the vehicle group) were decreased. Pretreatment with paeoniflorin reversed the effects on IL-1β (Tukey's tests, *P*<0.01) and TNF-α (Tukey's tests, *P*<0.05), but not the other cytokines. However, the IL-9 (Tukey's tests, *P*<0.001) level was further decreased in the paeoniflorin group and the escitalopram group than those in the vehicle group. Similarly, escitalopram reversed the elevated IL-1β (Tukey's tests, *P*<0.05) and TNF-α (Tukey's tests, *P*<0.05) levels. However, the level of IFN-γ (Tukey's tests, P<0.05) appeared to be higher than that in the vehicle control group (Figure 6).

### Paeoniflorin reverses the elevated density of microglia in the medial prefrontal cortex after IFN-α treatment

**Figure 7:**
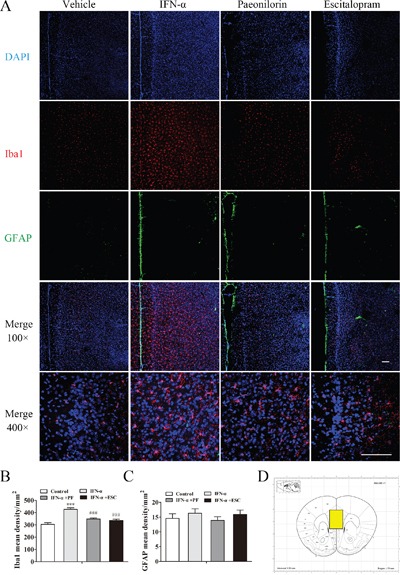
Paeoniflorin reduced interferon (IFN)-α-induced neuroinflammation in the medial prefrontal cortex Immunofluorescence co-staining of Iba1(red) and GFAP (green) after pretreatment with paeoniflorin (40 mg/kg i.g. daily for 4 weeks) and systemic IFN-α administration (15×106 IU/kg s.c.) for 4 weeks. Nuclei are counterstained with DAPI (blue). **A**. Quantification of the number of activated Iba1-and GFAP-positive cells after IFN-α treatment using an observer-independent unbiased stereology approach. **B, C**. Immunoreactivity was evaluated for the microglial marker Iba1 and the astrocytic marker GFAP in the region of interest. The elevated activated microglia in the medial prefrontal cortex of IFN-α-injected mice were reduced by both paeoniflorin and escitalopram (10 mg/kg). Astrocytes were not significantly affected by IFN-α in this region. The region of interest (the medial prefrontal cortex) is shown in the stereotaxic atlas of the mouse brain. **D**. Representative confocal microphotographs. Upper images: Iba1+GFAP+DAPI staining with an original magnification of × 100. Lower images:Iba1+GFAP+DAPI staining with an original magnification of × 400. Data are mean values ± SEM, evaluated by one-way analysis of variance followed by Tukey's post hoc tests (n=5 animals per group). *p< 0.05, **p< 0.01, ***p< 0.001 compared with the saline-treated group; #p< 0.05, ##p< 0.01, ###p< 0.001 compared with the IFN-α-treated group. Scale bar, 100 μm.

Because microglia and astrocytes are main sources of inflammatory signals in the brain, we investigated the responses of the microglial marker Iba1 and the astrocytic maker GFAP following paeoniflorin administration in the IFN-α-treated mPFC using immunofluorescence. Immunoreactivity to Iba1 significantly increased in the mPFC (for regions of interest, see Figure 7D) after 4 weeks’ exposure to IFN-α, and was pronouncedly reversed by high-dose paeoniflorin and escitalopram. Typical pictures are shown in Figure 7A. In contrast to Iba1, changes in the GFAP-positive cells failed to show significant effects (one-way ANOVA, *F*_3,16_=0.59, *P*>0.05 Figure 7C). Unlike microglia, astrocytes were found mainly at the brain surface, while microglia were found in all cortical layers. Interestingly, Iba1-positive cells were activated with rounded morphology after IFN-α treatment (one-way ANOVA, *F*_3,16_=20.58, *P*<0.001, followed by an post-hoc test, Tukey's tests, *P*<0.001, compared with the vehicle group), and these changes were reversed by high-dose paeoniflorin (Tukey's tests, *P*<0.001, compared with the IFN model group, Figure 7B).

### Paeoniflorin reverses the elevated densities of microglia and astrocytes in the ventral hippocampus after IFN-α treatment

**Figure 8:**
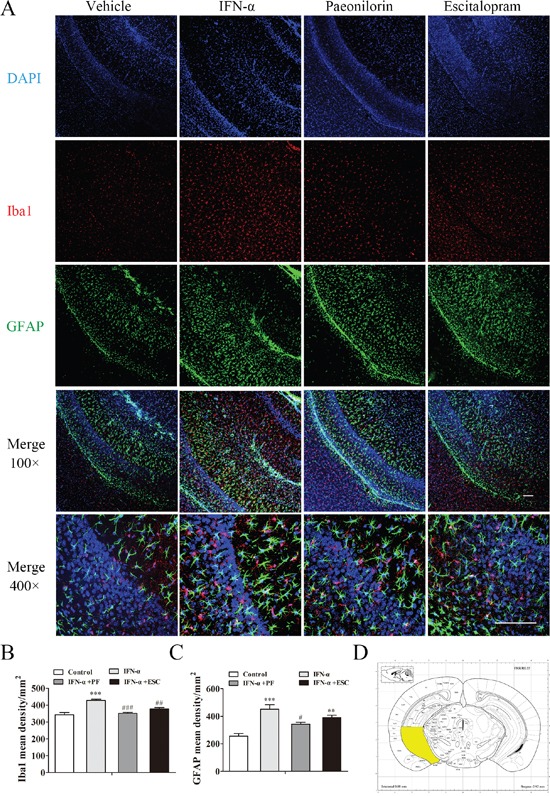
Paeoniflorin reduced interferon (IFN)-α-induced neuroinflammation in the ventral hippocampus Immunofluorescenceco-staining of Iba1(red) and GFAP (green) after pretreatment with paeoniflorin (40 mg/kg i.g. daily for 4weeks) and systemic IFN-α administration (15×106 IU/kg s.c.) for 4 weeks. Nuclei are counterstained with DAPI (blue). **A**. Quantification of the number of activated Iba1-and GFAP-positive cells after IFN-α treatment using an observer-independent unbiased stereology approach. **B, C**. Immunoreactivity was evaluated for the microglial marker Iba1 and the astrocytic marker GFAP in the region of interest. The elevated activated microglia and astrocytes in the ventral hippocampus of IFN-α-injected mice were reduced by both paeoniflorin and escitalopram (10 mg/kg). The region of interest (the ventral hippocampus) is shown in the stereotaxic atlas of the mouse brain. **D**. Representative confocal microphotographs. Upper images: Iba1+GFAP+DAPI staining with an original magnification of ×100. Lower images:Iba1+GFAP+DAPI staining with an original magnification of ×400. Data are mean values ± SEM, evaluated by one-way analysis of variance followed by Tukey's post hoc tests (n=5 animals per group). *p< 0.05, **p< 0.01, ***p< 0.001 compared with the saline-treated group; #p< 0.05, ##p< 0.01, ###p< 0.001 compared with the IFN-α-treated group. Scale bar, 100 μm.

The effect of paeoniflorin on Iba1- and GFAP-positive cells in the IFN-α-treated vHi was determined using immunofluorescence (for regions of interest, see Figure 8D). Iba1- and GFAP-positive cells were activated and recruited in this region following IFN-α treatment (Figure 8A). Immunoreactivity to Iba1 (one-way ANOVA, *F*_3,16_=16.52, *P*<0.001, followed by an post-hoc test, Tukey's tests, *P*<0.001, compared with the vehicle group, Figure 8B) and GFAP (one-way ANOVA, *F*_3,16_=13.75, *P*<0.001, followed by an post-hoc test, Tukey's tests, *P*<0.001, compared with the vehicle group, Figure 8C) was significantly increased and paeoniflorin reversed these changes (Tukey's tests, *P*<0.001, IFN model group vs. paeoniflorin group for Iba1; Tukey's tests, *P*<0.05, IFN model group vs. paeoniflorin group for GFAP). Escitalopram significantly reversed the changes in Iba1-positive cells (Tukey's tests, *P*<0.01) but not in GFAP-positive astrocytes (Tukey's tests, *P*>0.05), which were significantly increased compared with the control group. GFAP-positive astrocytes in the vHi were more abundant than the GFAP-positive cells in the mPFC, In addition, the volume of astrocytes was increased in IFN-α-treated mice, and this could not be reversed in the vHi by paeoniflorin (Figure 8).

### Paeoniflorin reverses the elevated density of microglia in the amygdale after IFN-α treatment

**Figure 9:**
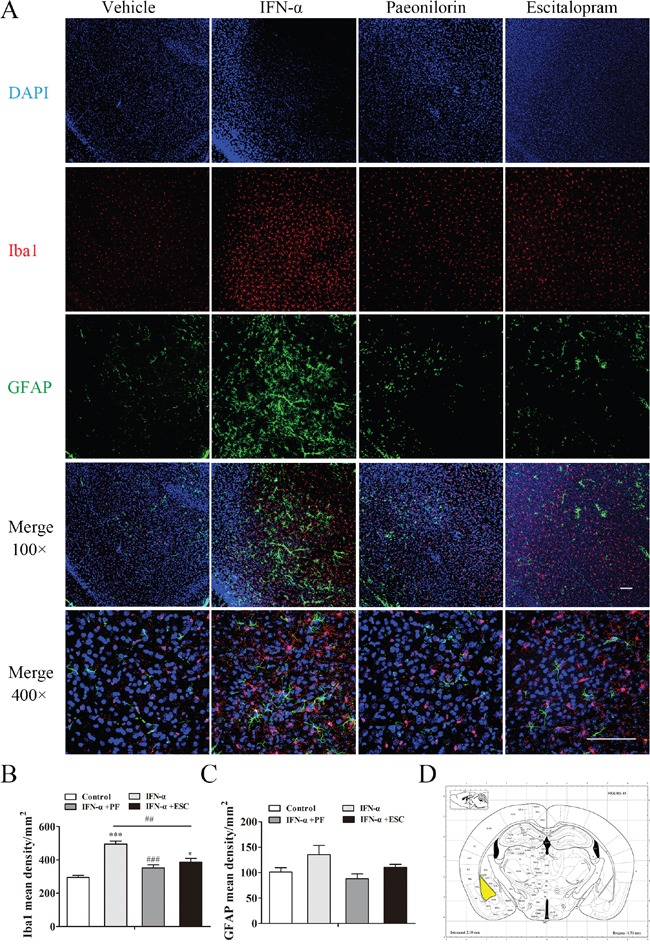
Paeoniflorin reduced interferon (IFN)-α-induced neuroinflammation in the amygdala Immunofluorescence co-staining of Iba1(red) and GFAP (green) after pretreatment with paeoniflorin (40 mg/kg i.g. daily for 4 weeks) and systemic IFN-α administration (15×106 IU/kg s.c.) for 4 weeks. Nuclei are counterstained with DAPI (blue). **A**. Quantification of the number of activated Iba1-and GFAP-positive cells after IFN-α treatment using an observer-independent unbiased stereology approach. **B, C**. Immunoreactivity was evaluated for the microglial marker Iba1 and the astrocytic marker GFAP in the region of interest. The elevated activated microglia in the amygdala of IFN-injected mouse were reduced by both paeoniflorin and escitalopram (10 mg/kg), although escitalopram was still associated with significantly increased levels compared with the saline-treated group. In addition, the volume of astrocytes was increased by IFN-α in this region. The region of interest (the amygdala) is shown in the stereotaxic atlas of the mouse brain. **D**. Representative confocal microphotographs. Upper images: Iba1+GFAP+DAPI staining with an original magnification of × 100. Lower images: Iba1+GFAP+DAPI staining with an original magnification of × 400. Data are mean values ± SEM, evaluated by one-way analysis of variance followed by Tukey's post hoc tests (n=5 animals per group). *p< 0.05, **p< 0.01, ***p< 0.001 compared with the saline-treated group; #p< 0.05, ##p< 0.01, ###p< 0.001 compared with the IFN-α-treated group. Scale bar, 100 μm.

The effect of paeoniflorin on Iba1-and GFAP-positive cells in the IFN-α-treated amygdala was examined using immunofluorescence (for regions of interest, see Figure 9D). Increased numbers of Iba1-positive cells were observed in the amygdala after systemic IFN-α treatment (one-way ANOVA, *F*_3,16_=21.65, *P*<0.001, followed by a post-hoc test, Tukey's tests, *P*<0.001, compared with the vehicle group, Figure 9A, 9B). Consistent with the other two regions, paeoniflorin (Tukey's tests, *P*<0.001, IFN model group vs. paeoniflorin group) and escitalopram (Tukey's tests, *P*<0.01, IFN model group vs. escitalopram group) reversed this significant change. Although immunoreactivity to GFAP was increased, this change failed to show any statistical significance (one-way ANOVA, *F*_3,16_=3.13, *P*>0.05, Figure 9A, 9C). In addition, the volume of astrocytes increased after IFN-α treatment; Paeoniflorin had no effect, while escitalopram decreased the astrocyte volume (Figure 9C).

## DISCUSSION

IFN-α is commonly used to treat infectious diseases and cancers. However, anhedonia and suicidal ideation are among the severe adverse effects associated with IFN-α therapy, especially with high-dose administration [2]. In the present study, pretreatment with paeoniflorin, prior to the systemic administration of IFN-α, significantly relieved both IFN-α-induced local neuroinflammation and depressive-like behaviors in mouse models. In particular, both the levels of inflammatory cytokines and the cytokine-associated microglia and astrocytes were changed in the emotion-related mPFC, vHi, and amygdala in a IFN-α-induced depression murine model, and paeoniflorin significantly reversed these changes compared with escitalopram. Interestingly, while the changes in microglia and astrocytes in the mPFC and vHi in our study are consistent with other studies [16, 42, 21], To our knowledge, this is the first study to demonstrate changes in local cytokines and the density of microglia and astrocytes in the emotion-related amygdala in an IFN-α-induced model of depression, suggesting amygdala is involved in the pathogenesis of IFN-α-induced depression.

IFN-α is known to be a proinflammatory cytokine and a potent inducer of the cytokine network [43]. Attention has been focused on the effects of cytokines of the innate immune system on the brain and behavior. The association between inflammation and depression was originally discovered in patients receiving long-term IFN-α treatment, and this association suggests that inflammation has an impact on emotional disorders or even acts as a potential pathogenic agent in depression [44, 45]. In terms of the immunologic mechanisms of IFN-α-induced depression, IFN-α directly acts on the central nervous system, as well as exerts indirect effects *via* the activation of other peripheral and central inflammatory cytokines. In our study, the changes of cytokines in peripheral and the brain were different. The levels of cytokines, except IL-6, decreased in peripheral but increased in the brain. It was suggested that the stimulation from IFN-α for a long time might lead to the cytokines which released from peripheral to get into the brain through the blood brain barrier and some of them might stimulate the brain to release more cytokines in a positive feedback manner. When the stimulation of IFN-α continued, the levels of cytokines in peripheral decreased when the body might adapt the stimulation or the cytokines have already entered the brain and hence increased the levels in the brain. Besides, the changes of IL-6 in peripheral was similar with that in the brain, which suggests that IL-6 is an important cytokines. Thus, in the current study, when we injected IFN-α systemically, IFN-α did not only change the peripheral cytokine network, but increased the local cytokines production in microglia, as shown by the elevated levels of the inflammatory mediators IL-6, IL-1β, and TNF-α in the mPFC, vHi, and amygdala [46]. Simultaneously, the stimulated microglia responded to these pathological inflammatory products with a reaction termed ‘microglial activation’ to maintain an immunologic balance in the brain [47]. However, ample evidence has shown that fully activated microglia cells are neurotoxic in the brain [48, 49, 47], which might have an association with the behavioral changes observed in the present study.

Cytokine-associated microglia and astrocytes are distributed in many brain regions. One of the major emotion-related brain regions is the mPFC, which has been reported that this region was associated with inflammation in depression; inflammation-related molecules that link repeated stress to mPFC dysfunction are potential targets of pharmaceutical development for psychiatric disorders [50, 51]. Furthermore, this region was thought to have a role in IFN-α-induced depression via activated microglia [16]. In the present study, microglia in the mPFC appeared to have an important role in the neuroinflammatory reaction in IFN-α-stimulated depression than astrocytes, and paeoniflorin appeared to mainly influence microglia, as opposed to astrocytes. That might be a result of the distribution of microglia and astrocytes in the mPFC. In addition, larger changes in cytokine levels were observed in the mPFC than in the vHi and amygdala after IFN-α administration. That observation might be associated with the increased MCP-1 level in the mPFC, which was not observed in the vHi and amygdala. MCP-1 can recruit peripheral immune cells in the brain, leading to additional cytokine and inflammatory mediator productions [46]. This explains why IL-10, an anti-inflammatory cytokine, was increased in both the mPFC and amygdala, but IL-9 and IL-12 levels were increased in the mPFC while decreased in the amygdala after IFN-α treatment. Paeoniflorin reversed most of the elevated cytokine levels in the mPFC of IFN-α-treated mice, suggesting that paeoniflorin has multiple effects on different cytokines and chemokines. Taken together, paeoniflorin has an excellent anti-neuroinflammatory effect in the mPFC by modulating activated microglia and cytokine release.

The vHi is believed to have a close relationship with the mPFC; a pathway from the mPFC to the vHi has a significant role in emotional memory processing [52]. Mounting evidence showing the close relationship between the vHi and emotion, with growing interest in the roles of vHi in depressive disorders [53, 18]. Many researchers have reported that the numbers of microglia and astrocytes, as well as their related cytokines, increase in the hippocampus of the IFN-α-treated brain [16, 42, 21]. The current results described changes in the vHi that are similar to those previously reported in the hippocampus. Compared with the mPFC and amygdala, not only the density of the microglia but also that of astrocytes was significantly changed in our IFN-α-induced depression mouse model, which could be a result of the ample distribution of both microglia and astrocytes in the vHi. However, fewer types of cytokines appeared to be increased in the vHi compared with the mPFC and amygdala. This indicates that astrocytes might play a role in the balance of cytokine release. Furthermore, paeoniflorin appeared to relieve neuroinflammation to a certain extent in the vHi although it was not associated with changes in the elevated IL-12 p70 and IFN-γ levels, which were similar to those observed with escitalopram.

The amygdala plays an important role in depressive disorders, being an upstream region to the vHi in social interactions and is associated with emotion-related gene expression in the vHi. It is reported that the major depressive disorder is associated with greater increases in inflammation in amygdala and reduction in amygdala volume that are thought to reflect dendritic atrophy which is associated with glia cells [54, 55]. However, little is known about the changes in the amygdala in IFN-α-induced depression. Our IFN-α-induced depression model showed significant changes in cytokine levels and activated microglia in this particular region after long-term IFN-α administration, which was similar to the changes in immune cells observed in the mPFC. Microglia in the amygdala showed more distinct changes in density than those in astrocytes did, suggesting that these cells are more sensitive to IFN-α stimulation compared to astrocytes. Furthermore, although the astrocyte density did not significantly change in the amygdala, there was an increasing trend. This suggests that microglia play an important role in response to IFN-α stimulation, while astrocytes play a complementary role. Paeoniflorin again showed certain anti-neuroinflammatory effects in the amygdala, reversed the increased levels of some cytokines and microglial density. Interestingly, the levels of IL-9 and IL-12 p70 significantly were decreased after long-term IFN-α administration, which is clearly different to the changes in the mPFC and vHi. This might be a result of the increased levels of IFN-γ and IL-10;with systemic IFN-α administration, type I IFN signaling was activated and produced the appropriate amount of IFN-γ [56, 57]. Cytokines associated with the type I immune response, such as IL-9, can inhibit type 2 immunity. In addition, microglia increased production of an anti-inflammatory cytokine IL-10, which can be stimulated by inflammatory cytokines to maintain the balance of the immune response in the brain. Recently, the IL-10 receptor has been reported to inhibit IL-12 release [57, 58]. Consequently, the levels of IL-9 and IL-12 p70 were decreased in the amygdala.

Escitalopram and paeoniflorin showed similar effects as antidepressants. Although escitalopram belongs to the class of SSRI antidepressants, it also exerted some anti-neuroinflammatory effects in this model and had some influence on activated microglia. Nevertheless, it had little influence on stimulated astrocytes, in the three emotion-related regions examined. However, escitalopram also possesses neuroinflammatory side effects, with a higher production of cytokines in the three brain regions compared with paeoniflorin and the non-IFN-α-treated group. About 30–50% of patients with major depressive disorder have been reported to fail to respond to approved antidepressant agents [45], which might also be associated with the described side effects. The evidence reported here suggests that paeoniflorin might be a suitable complementary agent for patients with IFN-α-induced depression because of its excellent anti-neuroinflammatory effects as well as its antidepressant effects in the major emotion-related regions. Nevertheless, paeoniflorin was seen to exert some side effects in this model, including increased levels of IL-12 and VEGF in the mPFC compared with the control group and decreased levels of IL-9 in the amygdala.

Our research provides more information on the different extents of inflammatory changes in emotion-related brain regions in mice systemically treated with IFN-α. In this article, we have described the anti-neuroinflammatory and antidepressant effects of paeoniflorin in a mouse model of chronic IFN-α-induced depression, with focus on the changes in immune cells and a series of cytokines in three emotion-related brain regions: the mPFC, vHi, and amygdala. Taken together, our findings indicate that paeoniflorin reversed the IFN-α-induced behavioral changes, probably by decreasing local cytokines and chemokines in some emotion-related regions and attenuating the numbers of microglia or astrocytes in these regions. In addition, we compared the therapeutic effects of paeoniflorin with escitalopram, which is commonly used in the clinic to treat patients with IFN-α-induced depression, and studied the anti-neuroinflammatory and antidepressant effects of both substances. This study provides solid scientific evidence to suggest paeoniflorin for clinical application in the treatment of IFN-α-induced depression.

Although the use of recombinant human IFN-α2b in establishing a murine model of IFN-α-induced depression remains controversial [59, 60], we found that the administration of high-dose recombinant human IFN-α2b over a long period did induce depressive-like behaviors and led to distinct neuroinflammatory changes in some emotion-related regions of the brain. Moreover, many studies have successfully established murine models of IFN-α-induced depression with human IFN to evaluate the clinical utility of this drug [38, 61–64]. Lastly, this study is limited by its relatively small sample size and the lack of analysis of the inflammation-associated mitogen-activated protein kinase signaling pathway. Except these closed related mPFC, vHi and amygdala, other regions may also associate with depression, such as striatum, we will study them in the future.

The results of our study suggest that long-term high-dose IFN-α treatment leads to locally distinct inflammation in periphery and neuroinflammation in the mPFC, vHi, and, in particular, the amygdala. Furthermore, paeoniflorin attenuated IFN-α-induced inflammation in serum and in these brain regions (which has not previously been described in detail) and ameliorated depressive-like behaviors in mice treated with long-term high-dose IFN-α. Paeoniflorin might therefore have therapeutic potential as a preventive agent for patients who need to receive long-term high-dose IFN-α treatment and who are susceptible to depression, and particularly in patients with inflammatory disorders. Further studies are needed to determine the precise mechanisms by which paeoniflorin ameliorates neuroinflammation and depressive behaviors. Our data suggest that paeoniflorin is a promising complementary drug for those patients who cannot receive SSRI antidepressant treatment.

## MATERIALS AND METHODS

### Animals

A total of 110 male, 8-week-old, C57BL/6Jmice (each weighing 23–25g) were purchased from Guangdong Medical Laboratory Animal Center, Guangzhou, China. All experimental protocols and the use of animals were approved by the Animal Care Ethics Committee of Southern Medical University (No.NFYY-2014-68) which follows People's Republic of China Laboratory Animal Regulations. All efforts were made to minimize the number and suffering of the mice. Mice were housed in a maximum of five mice per cage (30×20×15 cm) with food and water *ad libitum*. They were kept in 12h light/dark cycles (lights on at 07:00) at 23±1°C. The mice were let to habituate the experimental environment to reduce any stimulation arose from the new environment for a week before the experiment. At the end of this week, a sucrose preference test was conducted to record the baseline of sucrose preference percent of these mice without any operation and the mice who naturally not like sweet taste or the mice who have position preference were removed to reduce the individual difference. The mice then were let to have a rest and continue to habituate the experiment environment in the next week. At the end of this week, the weights of mice were recorded and groups were assigned randomly. All manipulations were carried out between 10:00 and 15:00. The researchers were blinded to the experimental conditions.

### Administration of IFN-α and paeoniflorin

A first set of mice was used to identify the dosage of IFN-α required to establish a model of IFN-α induced depression. These mice were given daily subcutaneous (s.c.) injections of either sterile saline solution or recombinant human IFN-α2b (Kawin Technology, Beijing, China), diluted with sterile saline, at doses from 0.06×10^6^ to 15×10^6^ IU/kg for 4 successive weeks [38]. A second set of mice was given IFN-α s.c. at a constant volume of 15×10^6^ IU/kg daily at every 10:30 for 4 successive weeks, followed by daily intragastric (i.g.) administration of paeoniflorin (purity 99%; Chengdu Must Bio-Technology Co., Ltd, China) at 10 mg/kg (low dose), 20 mg/kg (medium dose), or 40 mg/kg (high dose) dissolved in saline [30]; or escitalopram (10 mg/kg i.g.; H. Lundbeck, Denmark), 30 min before IFN-α administration. Appropriate vehicle (saline)-treated groups were also assessed simultaneously. The details of these two experimental designs are shown in Figures 1A and 2A.

### Behavioral assessment

Behavioral assessment was performed between 11:00 and 15:00 after week 4, when the administrations of IFN-α and paeoniflorin were ceased. Each testing session was arranged in the same order, with the researchers blinded to the experimental conditions.

### Sucrose preference test

A sucrose preference test (SPT) was performed as described previously [39]. In order to habituate mice to the sucrose solution, two bottles of 1% sucrose solution were placed in each cage for 24 h; at the same time, the mice were deprived of water. The SPT was carried out at 11:00–15:00. Consumption of sucrose solution and water was determined for 4 h. Each animal was given free access to two bottles, one contained 1% sucrose solution and the other contained water. For each group, the consumption of sucrose solution and water was measured by weighing the bottles. The relative amount of sucrose consumed was calculated using the following formula: sucrose consumption rate (%) = sucrose consumption/ (water consumption + sucrose consumption). The SPT was conducted 30 min following IFN-α administration.

### Open-field test

The open-field test was performed 24h after the SPT. The open-field apparatus consisted of a rectangular chamber (40×40×30 cm) made of gray polyvinyl chloride. The mice were gently placed in the center of the chamber and left to explore the area for 5 min. The digitized image of the path taken by each mouse was stored, and the locomotion activity and number of rearing were analyzed *post hoc* using EthoVision 7.0 software.

### Tail suspension test

The tail suspension test (TST) was performed 24h after the open-field test, as described previously [40]. Briefly, mice were suspended 35 cm above the floor in a visually isolated area by adhesive tape placed 1–1.5 cm from the tip of the tail. The test lasted for 6 min. The duration of immobility was recorded during the final 4 min by an investigator who was blinded to the study conditions. Immobility lasting for less than 2 s was not included in the analysis.

### Forced swimming test

The forced swimming test (FST) was performed 24 h after the TST, as described previously [41]. Briefly, the mice were placed in a vertical glass cylinder (35 cmheight×15 cm diameter) filled with 23 cm water at 23±1°C. The test lasted for 6 min. The duration of immobility was recorded during the last 4 min by a researcher who was blinded to the study conditions. Immobility was defined as only those movements required to keep the mouse afloat, and immobility lasting for less than 2 s was not included in the analysis.

### Cytokine array analysis

Twenty-four hours after the final behavioral assessment, 5 mice in each group were sacrificed by cervical dislocation after being anesthetized with chloral hydrate. The serum was collected, the brains were removed and tissue samples were collected from the vHi, mPFC, and amygdala, with reference from the stereotaxic atlas of the mouse brain. The Quantibody^®^ Mouse Cytokine Array 1 kit (RayBiotech, Norcross, GA, USA) was used according to the manufacturer's instructions to analyze the cytokines in the serum and brain tissues. Data extraction can be performed using the GAL file specific for this array, along with microarray analysis software (GenePix, ScanArray Express, ArrayVision, MicroVigene).

### Immunofluorescence

Immediately after the behavior assessments, 5 mice in each group were anesthetized and fixed by transcardiac perfusion with 4% paraformaldehyde in 0.1 M phosphate buffer. The brain was extracted, postfixed in the same fixative overnight, and cut into 40 μm coronal sections using a vibratome. The mPFC from each mouse was cut into 4 sections from bregma 1.98 to 1.42, the vHi was cut into 6 sections from bregma −2.92 to −3.52, and the amygdala was cut into 6 sections from bregma−0.94 to 1.82. For immunostaining, the sections were incubated for 1 hour in blocking solution (10% donkey serum and 0.2% Triton X-100 in phosphate-buffered saline) overnight at 4°C with the primary antibodies goat anti-ionized calcium binding adaptor molecule 1 (Iba1) (1:500; Abcam) and monoclonal mouse anti-glial fibrillary acidic protein (GFAP) (1:500; Cell Signaling Technology), and then for 1hour at room temperature with Alexa Fluor-conjugated secondary antibodies (1:500; Invitrogen). The sections were then washed with 0.05% Tween 20 in phosphate-buffered saline three times, for 15 min each. The sections were then stained with a 0.5μg/ml 4',6-diamidino-2-phenylindole (DAPI) (Cell Signaling Technology) staining solution for 20 min at room temperature and then washed. Samples belonging to the same group were acquired in parallel and with the same settings. Fluorescence microcopy images were obtained using confocal microscopy (Nikon C2+, Japan) with a 10× and 40× objective. Positive cells were quantified using Image J software. In all of the histologic analyses, the number of cells in every three 40-μm-thick coronal section was counted.

### Statistical analyses

SPSS 13.0 statistical software was used for statistical analyses. All of the results are presented as the means ± SEM. Potential differences between the mean values were evaluated using one-way analysis of variance followed by the Tukey's tests for *post hoc* comparisons where equal variances were assumed. A p-value of<0.05 was considered significant. Histograms were performed using the GraphPad Prism software (version 5.01).

## Abbreviations

FST: forced-swimming test
GFAP: glial fibrillary acidic protein
Iba1: ionized calcium binding adaptor molecule 1
IFN: interferon
i.g: intragastric
IL: interleukin
MCP: monocytechemoattractant protein
mPFC: medial prefrontal cortex
s.c: subcutaneous
SPT: sucrosepreference test
SSRI: selective serotonin reuptake inhibitor
TNF: tumor necrosis factor
TST: tailsuspension test
VEGF: vascular endothelial growth factor
vHi: ventral hippocampus
